# Sleep Valuation Is Associated with Components of Sleep Health and Daytime Functioning in a College Sample: A Survey Study

**DOI:** 10.3390/ijerph18115644

**Published:** 2021-05-25

**Authors:** Spencer A. Nielson, Jordan Taylor, Zach Simmons, Andrea N. Decker, Daniel B. Kay, Matthew R. Cribbet

**Affiliations:** 1 Department of Psychology, Brigham Young University, Provo, UT 84606, USA; spencernielson1801@gmail.com (S.A.N.); zsimmons0@gmail.com (Z.S.); daniel_kay@byu.edu (D.B.K.); 2 Department of Psychology, University of Alabama, Tuscaloosa, AL 35487-0348, USA; jataylor9@crimson.ua.edu (J.T.); andecker@gsuite.crimson.ua.edu (A.N.D.)

**Keywords:** sleep health, sleep valuation, daytime functioning

## Abstract

Sleep valuation, the worth individuals place on sleep, is an understudied construct in the field of sleep medicine. This study introduced a Sleep Valuation Item Bank and explored how sleep valuation is related to sleep health and daytime functioning within a sample of college students. The participants in this study were 247 (85% white, 83% female) undergraduate students who completed an online survey that included questions from a Sleep Valuation Item Bank and questions about sleep and daytime functioning. Correlation and regression analyses were conducted to determine associations between sleep valuation, aspects of sleep health and daytime functioning. Mediation analyses were conducted to determine whether the sleep health variables explained the associations between sleep valuation and daytime functioning. In correlation analyses, sleep valuation was negatively associated with sleepiness and sleep quality. It was also associated with daytime functioning, including general mental and physical health, depression, and anxiety. In the regression analyses, daytime impairments including poorer physical and mental health, anxiety, and depression were associated with higher sleep valuation. Poorer sleep health, including greater sleepiness and lower sleep quality, explained these associations and were associated with higher sleep valuation. Thus, while daytime impairments, such as anxiety and depression, are related to sleep valuation, this relationship may be due in part to the sleep disturbance that often co-occurs with these impairments.

## 1. Introduction

Sleep valuation is the worth an individual places on their sleep. The value placed on objects and activities can profoundly impact personal decisions and social policies related to those objects and events. The value placed on sleep, the reasons people value sleep, and how these values impact personal decisions about sleep activities are poorly understood. Prospect theory, a theory that describes the influence of outcomes on decision-making, has shown that whether an individual values a gain or the individual is averse to a loss directly impacts monetary decisions [1]. Conversely, value increases when a commodity is rarified. Thus, poor sleep health and sleep-related daytime impairment may increase sleep valuation. More broadly, health valuation has been shown to influence personal health behaviors [2,3]. Placing a higher value on one’s health is associated with preventative behaviors, such as good hygiene and promoting healthy lifestyles [2,4]. Sleep health is important to an individual’s physical and mental well-being and cognitive performance [5,6,7,8,9,10], which likely contributes to sleep valuation. However, many people may express higher value in sleep in ways that paradoxically reduce sleep health (e.g., spending more time in bed, sleeping in, worry about sleep). Sleep valuation may manifest in attitudes about sleep, such as whether individuals believe sleep is valuable for its own sake, whether sleep is valued for its benefits, or whether sleep is not valued. Sleep valuation could also manifest in an individual’s behavior, such as scheduling one’s day around sleep or prioritizing sleep over other activities. While monetary comparison questions may be useful in determining an individual’s level of sleep valuation (e.g., I would rather work an extra hour at minimum wage than sleep for another hour), sleep valuation is not a monetary value construct. Rather, sleep valuation is about an individual’s determination of how much value their sleep has in their life, and is manifest most notably in attitudes and behaviors. Currently, there are no measures available for sleep valuation. Exploring how sleep valuation relates to sleep health and daytime performance may lead to new approaches to targeting personal values to improve sleep health.

Sleep health is a “multidimensional pattern of sleep-wakefulness, adapted to individual, social, and environmental demands, that promotes physical and mental well-being” [5]. Dimensions of sleep health include sleep quality/satisfaction, alertness/sleepiness, sleep timing, regularity, sleep duration, rhythmicity, and sleep efficiency [5,11,12]. This conceptualization places sleep health on a continuum that quantifies sleep in terms of positive features [13]. Each sleep health domain is significantly associated with a wide range of health outcomes, including mortality, obesity, coronary heart disease, and impaired neurobehavioral performance [5,6,7,8,10,14,15,16,17,18,19,20,21,22,23,24]. Sleep is a multidimensional construct that can be measured across multiple levels of analysis and along multiple dimensions. A sleep health framework not only argues that sleep is a multidimensional construct, but that a combination of sleep characteristics is more likely to be associated with physical health outcomes rather than any individual outcome [25,26]. Indeed, several studies have shown that greater sleep health is associated with better physical and mental health outcomes while poorer sleep health is associated with poorer physical and mental health outcomes [11,25,26,27,28,29]. Moreover, poor sleep health is associated with impairments in daytime functioning, including poorer cognitive performance, increased sleepiness, poorer work performance, and drowsy driving [9,19,30,31,32,33,34,35]. Past research has also demonstrated the importance of sleep health for social functioning. The combination of poor sleep quality and short or long sleep duration is particularly detrimental to social functioning in college-aged and middle-aged adults [36,37,38]. Sleep deprivation may even lead to changes in the neural pathways that regulate aspects of social functioning, such as behavioral and emotional regulation and attention to social cues [39]. Aspects of sleep health, such as sleep quality, alertness/sleepiness, sleep duration, and sleep efficiency may be linked to sleep valuation. However, the relationship between sleep valuation, sleep health, and daytime functioning has not been previously studied.

Economic, survey, and questionnaire studies suggest that people vary widely in how much they value their sleep. Consumer sleep tracking devices are among the top sellers in the health market, and consumer sleep tracking apps are among the most downloaded apps [40]. Economists have estimated that the opportunity cost of sleep is $16.19 per hour [41]. In 1999, it was estimated that $13.93 billion was spent to treat insomnia in the United States, while another study, in 1995, estimated that $2.067 billion is spent to treat insomnia in France [42]. Others have estimated that the economic cost of inadequate sleep in Australia was $45.21 billion [43], while the economic cost of insomnia was estimated at $6.6 billion in Canada [44]. In terms of quality adjusted life-years, losses in sleep are more valued than losses in fatigue and social functioning [45]. Collectively, these findings suggest that many people place a high value on their sleep, or at least on bettering their sleep. On the other hand, there is evidence that modern society is experiencing a crisis of sleep devaluation. The Better Sleep Council found that 15% of Americans feel that sleep is wasted time, and only 14% of Americans viewed sleep as their favorite part of the day [46]. Furthermore, people are willing to sacrifice sleep health for other activities, including academic study, social engagement, media use, and entertainment [47,48,49]. This is especially prevalent in college students, a population of emerging adults known for poor sleep habits [50,51,52,53]. Between 36% and 70% of college students report getting less than 7 h of sleep per night and 60% classified as poor sleepers (PSQI < 5) [50,52]. Poor sleep in college students is associated with depression, anxiety, attention deficit hyperactivity disorder, and poorer self-rated physical and mental health [51,52,54,55]. Poor sleep in college students is also associated with deleterious effects on daytime functioning, such as poorer cognitive performance, poorer academic performance, lower GPA, and stress [51,53,54,55,56]. College is a transition period in many emerging adults’ lives, and lifestyle choices about health made in college may directly impact health habits in adulthood [4]. For example, college students make decisions about how much they will value their sleep among academic and social pressures to stay awake longer. Relating sleep valuation with aspects of sleep health and daytime functioning may help to promote healthy sleep among a population at particular risk for poor sleep [45,46,47,48,49,50].

This study sought to fill the gap in the literature by introducing a Sleep Valuation Item Bank. This study also explored how sleep valuation relates to dimensions of sleep health and daytime functioning in a college sample. Examining these associations in a sample of college students is important, as this population is not only faced with the daytime consequences of poor sleep health [52], but make many decisions about how to balance their sleep health with other valued activities including academic and social activities [57]. The data for this study were collected at the onset of the COVID-19 pandemic, a time of high stress and poorer mental health [58,59,60]. As such, these data may be especially salient as they explore how college students value their sleep in conditions of change and stress.

## 2. Materials and Methods

### 2.1. Participants

To assess sleep valuation in a college sample, a convenience sample of 247 participants were recruited from introductory psychology classes at the University of Alabama, a large Southeastern university. Participation fulfilled a course requirement. For ethical considerations, an alternative assignment was also available. Participants were also assigned credit regardless of survey completion. Participants were screened and excluded from the study if they had a diagnosed sleep disorder, engaged in shift work, or were currently pregnant. No participants met these exclusion criteria. One participant was removed from analyses listwise for not reporting income and 3 were removed listwise for not completing a depression questionnaire within the survey. Sample characteristics are presented in Table 1. In brief, the sample was predominately white (85% White, 7% African American, 2% Asian, 2% Hispanic, 5% Mixed race), female (82% female, 23% female, 1% other), and young adults (*M* = 19 ± 1 years of age).

### 2.2. Procedures

Participants completed an online survey via Qualtrics. They answered questions regarding their demographics (age, gender, race, marital status, education, and household income), sleep valuation, sleep habits, daytime sleepiness, symptoms of anxiety and depression, and self-rated physical and self-rated mental health. Participants indicated household income by selecting the range in which their household’s gross annual income fell (up to $10,000, $10,000–$40,000, $40,001–$90,000, $90,001–$190,000, and $190,000+). Finally, participants specified whether they were completing the questionnaire on a workday (70%) or a free day (30%). All subjects gave their informed consent for inclusion before they participated in the study. The study was conducted in accordance with the Declaration of Helsinki, and the protocol was approved by the Ethics Committee of University of Alabama (#19-06-2476).

### 2.3. Measures

#### 2.3.1. Sleep Valuation

Sleep valuation was assessed using a Sleep Valuation Item Bank developed by Dr. Kay and his sleep research team. This item bank included 43 questions that assessed attitudes and behaviors thought to indicate the value participants had for their sleep. Items were created to get at valuation of sleep for its own intrinsic value, sleep valuation based on the need for sleep or the negative consequences of losing sleep, and indications that sleep was not valued. Consultations with experts in the fields of sleep medicine and economics (*n* = 6) were also used to determine which items should be used to assess sleep valuation. Participants rated the strength of agreement with each item on the SVQ on a slider scale, 0 (strongly disagree) to 100 (strongly agree). The default slider position was set to 50. Individual items and their distributional properties in the sample are presented in Table 2. On review of the items, we eliminated 3 items due to their poor face validity (i.e., measuring depression). These items had been flagged by peer-experts as potentially problematic. Two of the peer reviewers noted that these items may reflect depression or mental health problems rather than sleep valuation. Although other items were flagged as potentially related to mental health or sleep disorders by peer-reviewers (Items 9, 15, 26, and 31), these 3 were the ones that our team unanimously agreed did not have good face validity. Due to the small number of peer-reviewers, future peer-review of items in the Sleep Valuation Item Bank is warranted. With the remaining items, a sleep valuation total score was computed by first recoding reverse scored items and then summing the items, with higher scores indicating greater sleep valuation.

#### 2.3.2. Sleep Health Variables

The Pittsburgh Sleep Quality Index (PSQI) [61] was used to assess sleep quality, sleep efficiency, and total sleep time over the past month. The PSQI is a widely used 19-item questionnaire with good internal consistency (*α* = 0.80) [62]. To get at the sleepiness dimension of sleep health, the Epworth Sleepiness Scale (ESS) [63] and the Karolinska Sleepiness Scale (KSS) [64] were used. The ESS [63] is an eight-item questionnaire that assesses general levels of daytime sleepiness [63]. The ESS asks participants how likely they would be to fall asleep in certain scenarios, such as sitting in a car or watching TV. Higher scores on the ESS correlate with higher levels of daytime sleepiness. The ESS has been shown to distinguish healthy sleepers from disordered sleepers [65,66] and yielded acceptable internal consistency in the current study (α = 0.79). The KSS measures current subjective level of sleepiness [64]. Participants are asked to indicate subjective sleepiness over the last five minutes using a nine-point scale: 1 = extremely alert, 9 = very sleepy, great effort to keep awake, fighting sleep. The KSS is highly correlated with electroencephalographic (EEG) and behavioral markers of sleepiness [67].

#### 2.3.3. Daytime Functioning Variables

Daytime functioning variables included self-reported mental and physical health, anxiety, and depression. Participants rated their physical and mental health using two separate visual analogue scales ranging from 0 to 100, with 0 indicating “extremely unhealthy” and 100 indicating “extremely healthy” on both scales. The default slider position was set at 50. Indices of self-reported general and mental health are robust predictors of mortality [68,69] and morbidity [70]. Anxiety was measured using the National Institutes of Health Patient-Reported Outcomes Measurement Information System (PROMIS) Anxiety Short Form. This anxiety measure is composed of 7 items that assesses general feelings of anxiety over the past week. Participants are presented with statements, such as “I felt fearful” or “I felt worried” and then asked to answer how frequently these feelings occurred over the past week. Items are rated on a five-point Likert scale (i.e., 1 = Never, 2 = Rarely, 3 = Sometimes, 4 = Often, and 5 = Always). The questionnaire has been shown to have good psychometric properties in large ethnically diverse samples [71]. The current study reported a high internal consistency (α = 0.95). Depression was assessed using the Center for Epidemiologic Studies Depression Scale (CES-D) [72]. This depression measure is a 20-item questionnaire that assesses six factors of depression (i.e., depressed mood, feelings of guilt and worthlessness, feelings of helplessness and hopelessness, psychomotor issues, loss of appetite, and sleep disorders). Participants are presented with statements, such as “I was bothered by things that usually didn’t bother me” and rate how often the indicated feeling or behavior occurred over the past week. Items are rated on a four-point Likert scale (i.e., 0 = Rarely or none of the time, 1 = Some of the time, 2 = Occasionally or a moderate amount of the time, and 3 = Most or all of the time). Higher scores on the CES-D correlate with greater feelings of depression. High internal consistency has been reported across studies [72], with the current study reporting an internal consistency of (α = 0.79).

#### 2.3.4. Preliminary Analyses

Internal consistency of the items of the Sleep Valuation Item Bank was determined using Cronbach’s alpha and item correlations with the total score. To test bivariate associations among study variables, correlations were conducted. Correlation coefficients were used to examine aspects of convergent and discriminant validity between sleep valuation, anxiety, depression, sleep quality, and daytime sleepiness. An independent samples *t*-test was conducted to determine if sleep valuation differed between free days and workdays.

#### 2.3.5. Main Analyses

Multiple regression analyses were conducted to investigate predictors of the sleep valuation total score derived from the Sleep Valuation Item Bank. Separate analyses were run to further assess the associations between sleep valuation and sleep quality, sleep duration, sleep efficiency, both measures of daytime sleepiness, anxiety, depression, and self-rated physical and mental health while controlling for age, gender, race, marital status, and income.

Four separate multiple mediation models were constructed to explore sleep quality and daytime sleepiness as mediators of the relationships between sleep valuation and depression, anxiety, self-reported physical health, and self-reported mental health. Maximum likelihood with missing values estimation was performed. All analyses were conducted in STATA 16 (StataCorp. 2019. Stata Statistical Software: Release 16. College Station, TX: StataCorp LLC). Power analyses were conducted for parallel mediation models using montecarlo simulations [73]. For a sample size of 247 participants, a power of 0.58 and 0.77 was obtained for two mediators in a parallel mediation model.

## 3. Results

All items in the Sleep Valuation Item Bank had Cronbach’s alpha of 0.89 and higher, suggesting sufficient internal reliability. We also found that the total score of items was significantly correlated with daytime sleepiness as assessed on the Karolinska Sleepiness Scale (*p* < 0.001), sleep quality (*p* < 0.001), the self-rated physical health (*p* = 0.002), self-rated mental health (*p* < 0.001), depression (*p* = 0.002), and anxiety (*p* < 0.001) scores. The sleep valuation total score did not significantly correlate with the Epworth Sleepiness Scale (*p* = 0.275) score. Sleep valuation total score did not significantly differ between those who took the survey on free days (*n* = 74) or on workdays (*n* = 173) (*t* = −1.40, *p* = 0.163).

In a multiple regression analysis of the sleep valuation total score with demographic variables as covariates (age, gender, race, marital status, education, and household income), we found that being female was associated with higher sleep valuation total scores (*t* = 4.04, *p* < 0.001) holding constant all other covariates. No other demographic variables were significantly associated with the sleep valuation total score. We then completed multiple regression analyses testing the association between the sleep valuation total score and each aspect of sleep health or daytime functioning measure controlling for demographic variables. We found that higher self-reported general health, general mental health, and overall sleep quality were significantly associated with lower sleep valuation. Further, we found that higher self-reported sleepiness, sleep disturbance, symptoms of depression, and symptoms of anxiety were significantly associated with higher sleep valuation (Table 3).

**Figure 1:**
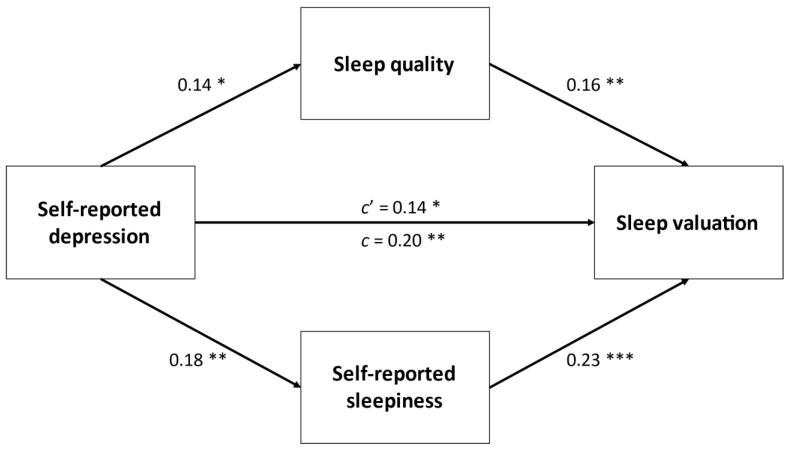
Parallel mediation between self-reported depression and sleep valuation. Values are standardized *β*. * *p* < 0.05, ** *p* < 0.01, *** *p* < 0.001.

**Figure 2:**
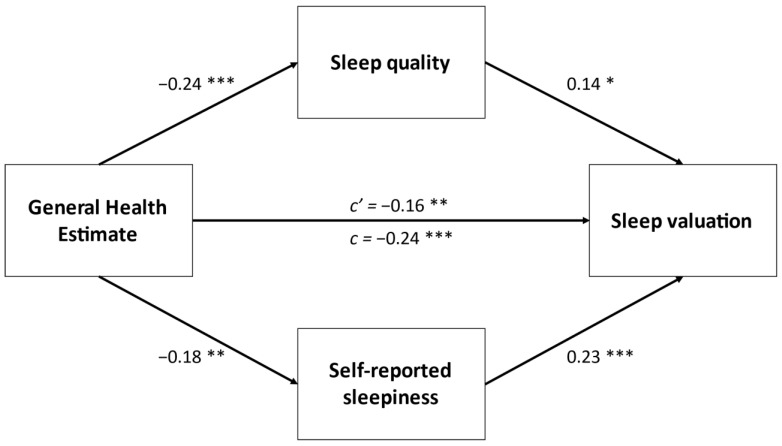
Parallel mediation between self-reported general health estimate and sleep valuation. Values are standardized *β*. * *p* < 0.05, ** *p* < 0.01, *** *p* < 0.001.

**Figure 3:**
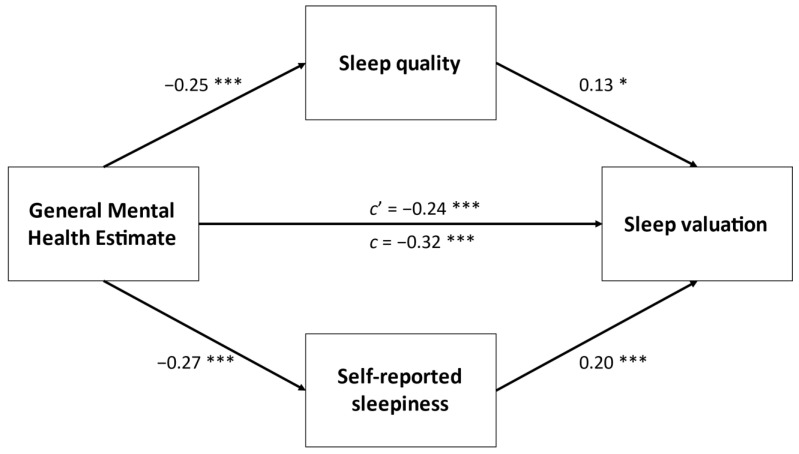
Parallel mediation between self-reported general mental health estimate and sleep valuation. Values are standardized *β*. * *p* < 0.05, *** *p* < 0.001.

**Figure 4:**
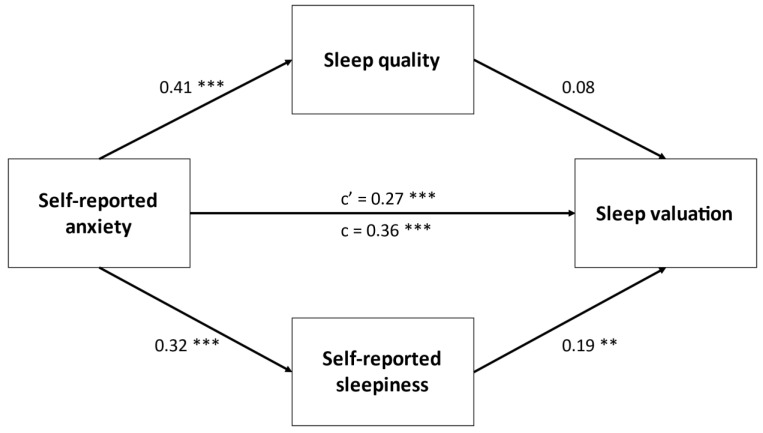
Parallel mediation between self-reported anxiety and sleep valuation. Values are standardized *β*. ** *p* < 0.01, *** *p* < 0.001.

Lastly, we conducted four separate mediation analyses with parallel mediators and found that overall sleep quality (PSQI item 6) and sleepiness partially mediated the relationship between self-reported depression (*p* = 0.003), general health estimate (*p* = 0.001), general mental health estimate (*p* = 0.001), self-reported anxiety (*p* = 0.004), and total sleep valuation score. Only self-reported sleepiness partially mediated the link between self-reported anxiety and sleep valuation. These results of these analyses are displayed in Figure 1, Figure 2, Figure 3 and Figure 4.

## 4. Discussion

The purpose of this study was to explore associations between sleep valuation and aspects of sleep health and daytime functioning in a sample of college students. Using a Sleep Valuation Item Bank, we found that increased daytime sleepiness, lower sleep quality, and greater daytime impairments including poorer physical and mental health, anxiety, and depression were associated with higher sleep valuation. Aspects of self-reported sleep health (self-reported sleepiness and sleep quality) partially explained the association between daytime functioning and sleep valuation. While an explanation of the underlying mechanisms for these associations requires further research, these results suggest that while daytime impairments, such as anxiety and depression, are related to sleep valuation, this relationship may be due in part to the sleep disturbance that often co-occurs with these impairments.

Although sleep valuation is an understudied construct in the field of sleep health, a couple of nationally representative surveys in the United States (U.S.) have collected data from questions that reflect the construct of sleep valuation. In one such survey, many American adults (82%) reported that one extra hour of sleep at night would be “somewhat or extremely valuable” indicating an increased valuation for sleep when not getting enough sleep [74]. Further, this same survey found that although 82% of Americans reported one extra hour of sleep as valuable, only 26% of Americans reported that they “would choose sleep over other activities if they were given an extra hour in the day” [74]. Another such survey found that almost half of Americans report not getting enough sleep but “less than half of them take any one specific action to help them get better sleep” [45]. These findings could help explain the results of the current study, wherein those who value sleep more had poorer sleep health, as participants in the current study may have reported that sleep is valuable while also choosing other activities over sleeping, thus leading to poorer sleep health. We failed to find an association between sleep valuation and workdays versus free days. This conflicts with a previous survey that reported that Americans value sleep less on weekends [45]. One possible explanation for this discrepancy is that students may value their sleep differently across work/free days than other populations.

The finding that higher sleep valuation was associated with lower sleep health is paradoxical to much of the wider health valuation literature, where higher health valuation is generally, but not always, associated with more positive health behaviors [3,4,45]. One potential explanation for these findings could be that some individuals who value sleep may behave in ways that they think promote good sleep when in fact these behaviors are poor sleep habits. For example, individuals who value sleep more may spend more time in bed, try to catch up on sleep, take naps, or worry about their sleep. These types of reactions to poor sleep have been widely recognized as maladaptive perpetuating factors to continued poor sleep and insomnia [72]. Due to limited knowledge, those who value sleep may develop dysfunctional beliefs and attitudes about sleep that worsen sleep health. Various research using the Dysfunctional Beliefs About Sleep Scale, wherein participants self-report various beliefs and attitudes they have about sleep, has demonstrated associations between dysfunctional beliefs about sleep and poor sleep [75,76,77,78,79]. Moreover, the association between dysfunctional beliefs about sleep and poor sleep, particularly worse sleep quality, has been frequently observed in college students, even during this pandemic period [78,80,81,82]. As this study found that sleep valuation and sleep quality are associated, there could be associations between sleep valuation, dysfunctional beliefs about sleep, and aspects of sleep health, particularly sleep quality. Further research is necessary to investigate these associations. Investigating how sleep valuation and dysfunctional beliefs about sleep are associated could provide novel ways to improve sleep health among college students and in the general population more broadly. Importantly, such improvements may benefit physical and mental health and social functioning [11,25,26,27,28,29,36,37,38].

This study had limitations. First, this study was a cross-sectional study based on self-report data. As such, we are unable to make inferences about the causal relationships between sleep valuation and aspects of sleep health and daytime functioning. A second limitation of the present study is that the Sleep Valuation Item Bank requires further research for items to be fully validated and converted into a questionnaire. However, this study was able to demonstrate that the items in the Sleep Valuation Item Bank had sufficient internal reliability and high face validity, thereby providing the first steps needed for validation. Third, this sample was comprised of healthy, young, and largely female Caucasian university students. While sleep is clearly important in the lives of young adults [49,54], these findings should be replicated across the lifespan and in more diverse groups. The finding that female students valued their sleep more than male students should be interpreted with caution due to the relatively small number of males in the study. Nevertheless, in a survey conducted in 2014 by the Better Sleep Council, women were also found to value sleep more than men [45].

## 5. Conclusions

These limitations notwithstanding, this study was the first study to introduce a Sleep Valuation Item Bank and to associate sleep valuation with components of sleep health and daytime functioning. The results of the present study demonstrate that sleep valuation is associated with daytime functioning and that these results are partially explained by two components of sleep health, sleep quality and daytime sleepiness. Understanding the extent to which people value their sleep has clear implications for cognitive and behavioral sleep treatments, and the theoretical models that underlie the development of sleep disorders, such as insomnia disorder. Beyond the clinical implications stated above, a better understanding of why, and the extent to which, people value their sleep has clear public health implications, and could lead to novel approaches for promoting sleep health among the general population. Educating society on how to value sleep may be as important as encouraging society to value sleep.

## Figures and Tables

**Table 1:** Demographic characteristics.

Age, y	19 (1)
**Gender**	
Male	43 (17%)
Female	203 (82%)
Other	1 (<1%)
**Race**	
White	209 (85%)
Black/African American	17 (7%)
Asian/Asian American	4 (2%)
Hispanic/Latino	5 (2%)
Mixed race	12 (5%)
**Marital Status**	
Married	1 (<1%)
Never married	238 (96%)
Cohabitating	8 (3%)
**Education**	
Graduated high school	115 (47%)
Graduated high school equivalent	21 (9%)
Some college	107 (43%)
Graduated 2-year college	1 (<1%)
Graduated 4-year college	3 (1%)
**Household Income**	
<$10,000	17 (7%)
$10,000-$40,000	15 (6%)
$40,001–$90,000	55 (22%)
$90,001–$190,000	98 (40%)
>$190,000	61 (25%)

Note. Means and standard deviations are reported as *M(SD)* and sample size and % of sample are reported as *n* (%).

**Table 2:** Sleep Valuation Item Bank.

#	Item	Mean	SD	Range	Reverse Scored	Skewness	Kurtosis	*α*	Item-Total Correlation
1	I generally desire more sleep	80.17	18.39	19–100		−0.89	3.50	0.89	0.51
2	When I have nothing to do, I would prefer to sleep	60.06	28.30	0–100		−0.28	2.07	0.89	0.74
3	I put off going to sleep at night, even when I am sleepy	51.42	30.24	0–100	Yes	−0.02	1.82	0.89	0.06
4	I generally prefer to sleep in	75.70	25.69	0–100		−1.07	3.34	0.89	0.44
5	When I wake up in the morning or my alarm goes off, I generally try to go back to sleep	61.23	29.74	0–100		−0.43	2.07	0.89	0.36
6	If I had to choose between sleep in a little longer or eating breakfast, I would choose to sleep in	73.72	30.44	0–100		−1.08	3.01	0.89	0.40
7	I try to sleep as much as I can	73.11	23.67	2–100		−0.75	2.87	0.89	0.55
8	I take every opportunity I can to sleep	53.59	30.22	0–100		0.07	1.86	0.89	0.72
9	I would rather stay asleep than wake up	63.45	28.62	0–100		−0.44	2.18	0.89	0.65
10	I would rather get in bed to sleep at night than stay up to do household tasks	66.75	26.28	0–100		−0.40	2.25	0.89	0.66
11	I would rather get in bed to sleep at night than stay up later to do my hobbies	51.77	27.06	0–100		0.05	2.20	0.89	0.67
12	I would rather get in bed to sleep at night than stay up to engage in social activities	43.58	28.66	0–100		0.41	2.22	0.89	0.60
13	I avoid doing things that might disrupt my sleep	49.41	25.13	0–100		0.20	2.35	0.89	0.48
14	Sleep is less pleasant than being awake	65.07	22.53	0–100	Yes	−0.32	2.92	0.89	0.41
15	I want to sleep more even when I feel rested	46.36	27.39	0–100		0.28	2.21	0.89	0.61
16	When I’m sleep, I would rather surf the web, watch a movie, engage in social media, or play a video game than go to sleep	50.56	27.45	0–100	Yes	0.08	2.10	0.90	0.11
17*	I want to sleep because I don’t like being awake	26.14	26.77	0–100		0.91	2.77	0.89	0.48
18	I would want to sleep more if my sleep were more restful	49.45	25.84	0–100		−0.04	2.44	0.89	0.18
19	I want to sleep more because I enjoy it so much	55.96	25.14	0–100		−0.08	2.53	0.89	0.72
20	When I feel sleepy at night, I try to go to sleep	71.60	22.89	2–100		−0.70	2.88	0.89	0.25
21	When I feel sleepy at night, I push through it so I can stay awake longer	56.59	25.90	0–100	Yes	−0.20	2.17	0.90	0.21
22	I don’t mind feeling sleepy during the day	70.74	24.46	0–100	Yes	−0.70	2.63	0.90	0.07
23	I want to sleep more because I feel sleepy	67.80	24.44	0–100		−0.84	3.37	0.89	0.60
24	I would rather sleep an extra hour than work an extra hour at my current rate of pay (or if not currently employed, I would rather sleep an extra hour than work an extra hour at minimum wage)	54.57	30.63	0–100		−0.19	2.00	0.89	0.49
25	I would rather sleep an extra hour than spend an extra hour doing the things I enjoy	37.90	25.45	0–100		0.42	2.69	0.89	0.59
26	I want to sleep even when I am not sleepy	39.52	27.68	0–100		0.41	2.37	0.89	0.65
27	I want to sleep more because I am not getting enough restful sleep	57.83	27.36	0–100		−0.30	2.32	0.89	0.40
28	If I can’t sleep, I would rather take a sleep aid than remain awake	51.28	32.71	0–100		−0.15	1.75	0.89	0.45
29	I wish I could sleep less	67.89	25.88	0–100	Yes	−0.57	2.46	0.90	0.12
30 *	I dread waking up in the morning	59.69	29.63	0–100		−0.46	2.23	0.89	0.51
31	I dread going to sleep at night	75.94	23.03	1–100	Yes	−0.69	2.50	0.89	0.10
32 *	I want to sleep my life away	24.17	28.02	0–100		1.17	3.33	0.89	0.55
33	If I could function without sleep, I would sleep less than I do	46.36	31.45	0–100	Yes	0.13	1.85	0.90	0.15
34	I’m likely to wake up earlier than usual to do something I look forward to	28.81	25.48	0–100	Yes	1.04	3.45	0.89	0.30
35	I look forward to going to sleep	72.05	20.61	0–100		−0.44	2.93	0.89	0.66
36	I schedule my day around my sleep	33.43	26.72	0–100		0.64	2.66	0.89	0.51
37	I modify my daytime activities to accommodate my sleep	36.41	27.50	0–100		0.37	2.15	0.89	0.50
38	I keep track of how much sleep I’ve lost and how much sleep I hope to make up later	33.90	28.95	0–100		0.63	2.40	0.89	0.27
39	If I lose sleep on one night, I try to make it up by napping or sleep more another night	56.43	29.22	0–100		−0.53	2.20	0.89	0.44
40	If I need more sleep, I am likely to sleep in even if that means I will be late	30.17	29.20	0–100		0.70	2.36	0.89	0.40
41	I enjoy sleeping	82.12	18.02	4–100		−0.95	3.83	0.89	0.61
42	I feel that sleep is a waste of time	72.19	25.66	0–100	Yes	−0.65	2.45	0.89	0.34
43	I try to catch up on sleep on days off	72.00	22.72	0–100		−0.92	3.76	0.89	0.52

Note. * Items that were removed for poor face validity.

**Table 3:** Self-report measures as predictors for sleep valuation.

Measure	Correlation	*β*	*t*	95% *CI*	*p*
General Health Estimate	−0.24 ***	−6.18	−3.12	−10.07–−2.28	0.002 **
General Mental Health Estimate	−0.32 ***	−5.40	−4.54	−7.74–−3.06	<0.001 ***
Karolinska Sleepiness Scale (KSS)	0.30 ***	55.27	3.86	27.02–83.51	<0.001 ***
Pittsburgh Sleep Quality Index (PSQI)	0.35 ***	40.08	5.37	25.39–54.78	<0.001 ***
Center for Epidemiologic Studies Depression Scale (CES-D)	0.20 **	7.50	2.50	1.58–13.42	0.013 *
PROMIS Emotional Distress–Anxiety	0.36 ***	16.86	5.19	10.46–23.27	<0.001 ***
Overall sleep quality (PSQI item 6)	0.24 ***	133.83	3.42	56.69–210.97	0.001 **

Note. This model adjusts for age, gender, race, marital status, education level, and income. * *p* < 0.05, ** *p* < 0.01, *** *p* < 0.001.

## Data Availability

Data available upon request from the corresponding author.
